# Evaluation of cardiovascular fall risk assessment and managment in Dutch fall clinics comparing current practice to guideline recommendations

**DOI:** 10.1007/s41999-026-01481-3

**Published:** 2026-06-02

**Authors:** A. C. Pronk, R. Schroten, L. J. Seppala, E. P. van Poelgeest, B. A. Damoiseaux-Volman, J. A. H. R. Claassen, F. Mattace-Raso, S. C. de Ruiter, J. O. Daal, L. A. R. Zwart, T. N. Bongers, G. S. Spronk, S. M. Driessen, N. van der Velde

**Affiliations:** 1 Internal Medicine, Section of Geriatric Medicine, Amsterdam UMC Location University of Amsterdam, Meibergdreef 9, Amsterdam, The Netherlands; 2 Amsterdam Public Health, Aging and Later Life, Amsterdam, The Netherlands; 3 Department of Medical Informatics, Amsterdam UMC Location University of Amsterdam, Amsterdam, The Netherlands; 4 Department of Geriatric Medicine, Radboudumc, Nijmegen, The Netherlands; 5 Department of Cardiovascular Sciences, University of Leicester, Leicester, UK; 6 Department of Internal Medicine, Division of Geriatrics, Erasmus MC University Medical Center, Rotterdam, The Netherlands; 7 Department of Geriatric Medicine, Northwest Clinics, Alkmaar, The Netherlands; 8 Department of Geriatric Medicine, Dijklander Hospital, Hoorn, The Netherlands; 9 Department of Geriatric Medicine, Slingeland Hospital, Doetichem, The Netherlands; 10 Department of Geriatric Medicine, Gelre Hospitals, Apeldoorn, The Netherlands

**Keywords:** Geriatrics, Accidental falls, Older adults, Cardiovascular disorders, Fall clinics

## Abstract

**Background:**

Cardiovascular disorders are important fall risk factors, especially in unexplained recurrent falls. According to (international) guidelines, a standard workup of cardiovascular fall risk factors is indicated; however, its implementations is lagging. This study aimed to map the daily practice of cardiovascular fall risk assessment and management in Dutch fall clinics, comparing guideline recommendations (work-as-imagined) with actual practice (work-as-done), and to identify barriers to implementation.

**Methods:**

The functional resonance analysis method (FRAM) was used to visualize work-as-imagined and work-as-done. Input for work-as-imagined was the Dutch guideline on fall prevention, which follows the ESC syncope guideline recommendations where applicable. Work-as done and barriers for guideline implementation were derived from semi-structured interviews with Dutch fall clinic professionals.

**Results:**

Fourteen participants from eight fall clinics participated. Work-as-done related to the initial assessment of cardiovascular fall risk factors (e.g., ECG recording), was in line with work-as-imagined and implemented across the participating fall clinics. For the implementation of further cardiovascular assessments (e.g., head-up tilt testing), significant variability was observed across fall clinics, resulting in four different work-as-done FRAM models. The variations were mainly driven by the following barriers: unavailability of staff, gaps in knowledge, lack of expertise, and lack of resources.

**Conclusion:**

This study highlights important discrepancies between recommended cardiovascular fall risk assessment protocols and current practices in Dutch fall clinics. A structured care pathway could help standardize and optimize cardiovascular fall risk practices. Addressing the identified barriers to implementation and development in standardized care pathways could facilitate the implementation of the care pathway and optimize cardiovascular fall prevention practices in older adults.

**Supplementary Information:**

The online version contains supplementary material available at https://doi.org/10.1007/s41999-026-01481-3.

## Key summary points

**Aim:**

To map the cardiovascular fall risk assessment and management in Dutch fall clinics by comparing guideline recommendations with actual practice and identifying implementation barriers.

**Findings:**

The initial assessment of cardiovascular fall risk factors, was in line with guideline recommendations and implemented across the participating fall clinics. For the recommended further cardiovascular assessments significant variability was observed across the clinics, resulting in four different variants.

**Message:**

This study highlights the discrepancies between recommended cardiovascular fall risk assessment protocols and daily practice in Dutch fall clinics, suggesting that developing a structured care pathway, while addressing implementation barriers, could optimize cardiovascular fall risk assessments in older adults.

**Supplementary Information:**

The online version contains supplementary material available at https://doi.org/10.1007/s41999-026-01481-3.

## Introduction

In older adults, falls are a major public health problem leading to significant morbidity and mortality [1]. In 2017, 8.4 million older adults in Western Europe required medical treatment for fall-related injuries [2]. According to the World Health Organization, falls are the second leading cause of unintentional deaths, and for individuals aged 70 years and older falls are the predominant cause of injury-related deaths [3]. Cardiovascular disorders (CVD), such as orthostatic hypotension (OH), arrhythmias, carotid sinus hypersensitivity (CSH), and vasovagal syncope (VVS) are an important cause of non-accidental and unexplained falls [1, 4]. These conditions may lead to episodes of transient, recurrent cerebral hypoperfusion, resulting in imbalance, dizziness, falls, and/or temporary loss of consciousness (syncope) [5].

Distinguishing between syncope and falls can be challenging, as syncope often presents atypically in older adults. Eyewitnesses are frequently lacking, and older individuals may suffer from retrograde amnesia following a syncopal event. As a result, patients with syncope may present with falls [6–10]. Therefore, current guidelines underline the importance of a cardiovascular (CV) evaluation in the assessment and management of fall risk [1, 5]. This initial CV assessment should include a detailed history taking, physical examination, supine and standing blood pressure and heart rate measurements, and a 12-lead electrocardiogram (ECG). Depending on clinical indication and available resources, further CV assessments, such as echocardiography, head-up tilt test (HUTT), and/or carotid sinus massage (CSM) are recommended [1].

Despite these recommendations, there is significant variability in the content of CV assessments being used in fall clinics. Furthermore, older adults with falls and/or syncope are often evaluated across different specialties, such as cardiology, neurology, geriatrics, and emergency departments, which can lead to fragmentation and inconsistency in diagnostic tests [11]. A protocol-based, yet locally adaptable approach (care pathway) to systematically assess CV fall risk factors when performing a multifactorial fall risk assessment could help reduce this variability. Such a pathway could enhance diagnostic accuracy and reduce hospital admissions, healthcare resource consumption and overall healthcare costs [12, 13]. To develop a care pathway, it is necessary to have a clear view on the current practices within fall clinics. In this qualitative study, we aimed to map the current care provided in different Dutch outpatient fall clinics and compare these with guideline recommendations. Additionally, we aimed to identify barriers, potential facilitators and novel practices in the implementation of the recommended CV assessments in fall clinics countrywide.

## Methods

The methods and results are reported in line with best practices for qualitative research. The consolidated criteria for reporting qualitative research (COREQ) checklist [14] for this project can be found in Appendix 1 of the supplementary data section.

### Design

Between June and December 2022, we conducted semi-structured interviews focusing on the daily practice of CV assessments and management within a multifactorial fall risk assessment. A waiver for an ethical review was obtained from the ethics committee (for this project, approval under the act medical research involving human subjects (Dutch: WMO) was not required). Prior to each interview, written informed consent was obtained and data were anonymized.

### Setting and participants

The setting of this study was Dutch geriatric outpatient (fall) clinics. Interviews were conducted with medical specialists (geriatricians and internist-geriatricians) working at these clinics. The fall clinics were identified through a short survey distributed to the Dutch Special Interest Group on fall prevention, to which all Dutch fall clinics are affiliated. This survey included questions about the organizational structure of the fall clinics, the CV assessments included in their multifactorial fall risk assessment, and the availability of additional diagnostic tests within their respective departments. Following this, a random selection of fall clinics was made, including both university and community hospitals, as well as fall clinics with and without syncope care. Respondents were then invited to participate by email. We continued inviting respondents until data saturation was reached, which was defined as the point at which no new insights were gained regarding the research question [15].

### FRAM

The Functional Resonance Analysis Method (FRAM) was used as a structured method to visualize discrepancies between work-as-imagined (Dutch guidelines on fall prevention, which follows the ESC syncope guideline recommendations) and work-as-done (actual daily practice) in the context of the CV fall risk assessment [5, 16].

**Fig. 1:**
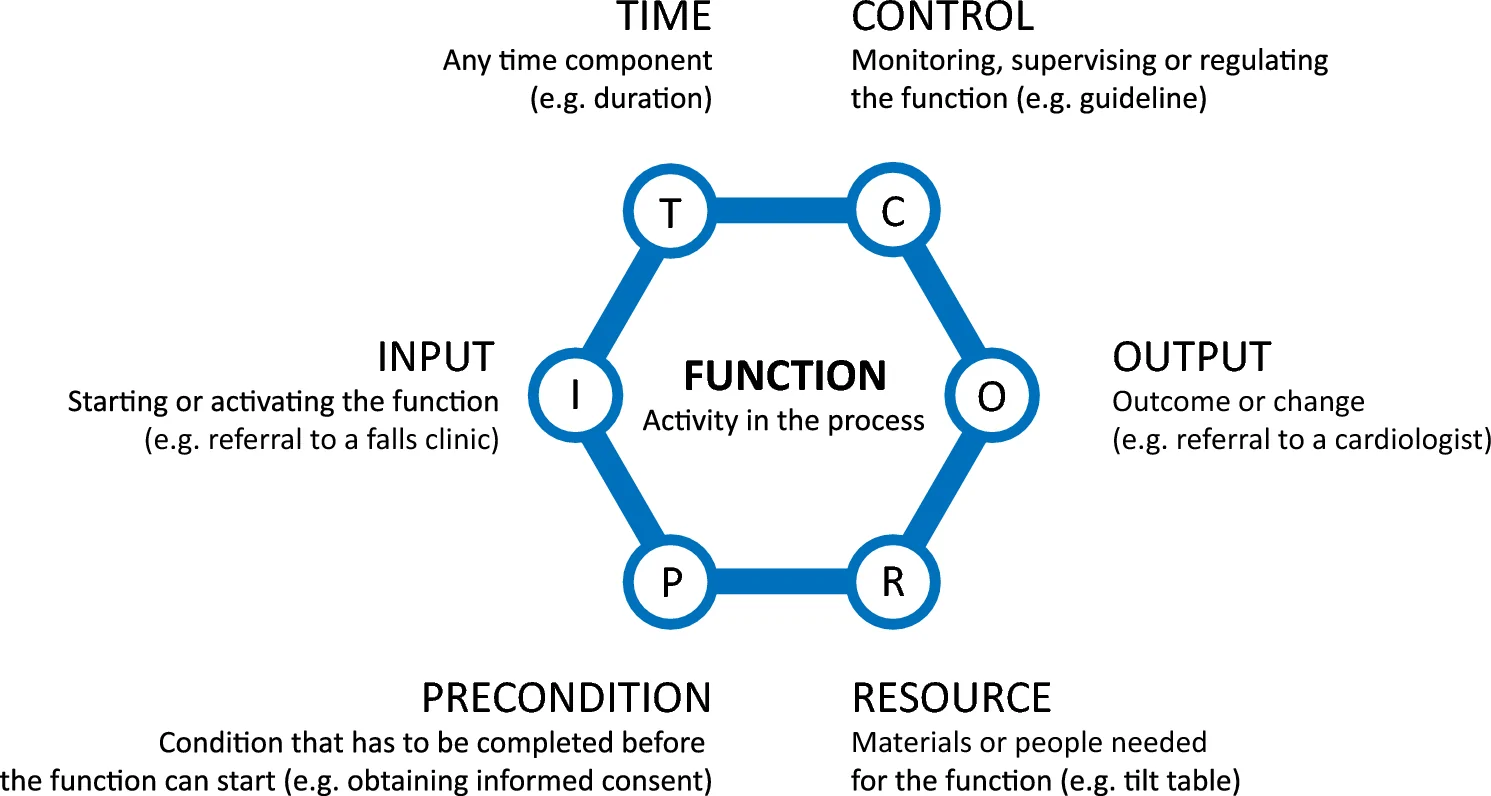
Example of a FRAM function [18]

FRAM was selected because it is particularly useful for analyzing and representing complex processes characterized by non-linear interactions. The method allows for the exploration and visualization of how different factors within a system interact and influence each other, while also accounting for the variability that characterizes routine practice [17, 18]. Unlike more conventional analytical approaches, FRAM does not rely on predefined models to describe system behavior [ref]. Instead, the method support the development of a model that identifies the functions contributing to overall system performance and the interdependencies between these functions [17]. In a FRAM model, the activities that make up the process are displayed as functions, visualized as hexagons. These functions are described by a verb (for instance *perform multifactorial fall risk assessment*) and described through six aspects: input, time, control, output, resource, and precondition (Fig. 1) [19].

In the FRAM model, if the output of one function matches the input of another function, a connection (line) is formed between the functions, allowing them to be linked. These functions, aspects, and their connections make up the FRAM model [17, 19]. By constructing separate FRAM models for work-as-imagined and work-as-done, differences between the two can be identified [17]. Functions in both work-as-imagined and work-as-done models were categorized into ‘foreground’ and ‘background’ functions. Foreground functions represent the main activities related to the CV fall risk assessment, while background functions encompass tasks and activities related to the process. A FRAM analysis comprises five key steps: (1) identifying the purpose of the analysis, (2) defining the system functions, (3) characterizing the variability of these functions, (4) mapping the interconnections among functions, and (5) determining potential outcomes and opportunities for process improvement [18]. The final step may aim to formulate recommendations for process enhancement, support innovation, or guide system redesign. In our study, the goal of this step was to identify barriers and facilitators regarding implementation of CV fall risk assessment within a multifactorial fall risk evaluation.

### Data collection

The FRAM model for work-as-imagined was based on the Dutch national guidelines on fall prevention [16], with particular emphasis on the chapter focusing on CV causes for falls, which was among others based on the European society of cardiology (ESC) guidelines for syncope [5]. The FRAM models for work-as-done were constructed from the data collected through the semi-structured interviews. Example of the interview guide is provided in Appendix 2 in the supplementary data section. The interview questions were based on the FRAM model for work-as-imagined. In addition, questions about potential barriers for implementing CV assessments in clinical practice, facilitators, and novel practices (diagnostic tests/or assessments not part of guidelines recommendations) were explored.

The interview questions were drafted by two researchers (AP, RS). The first researcher (AP) has a background in internal medicine and geriatrics. The second researcher (RS) was a student doing a research internship. The interview questions were then discussed and finalized by the research team (LS, EP, NV). A pilot interview was conducted with an independent geriatrician to optimize the interview guide. The interviews were conducted by two researchers (AP and RS). If there were questions or uncertainties about the CV assessment and management after the first interview, a second interview was held. All interviews were audio recorded, with the permission of the participants.

### Data analysis

All interviews were verbatim transcribed and manually coded independently by two coders (AP and RS). An initial code tree was constructed based on the functions and aspects of the FRAM model for work-as-imagined (Supplementary Table 1). The results of the coding were discussed between the two researchers until consensus was reached. An inductive approach (meaning the themes emerged from the data itself without being pre-defined by existing frameworks [20]) with open coding was used when answers from the participants did not fit the initial code tree, as well as for barriers, facilitators, and novel practices. MAXQDA 2020 (VERBI software) was used for coding and analyzing the data. Transcripts were not returned to the participants for their review. The FRAM models were first constructed using the FRAM Model Visualizer software (FMW version 2.1, September 2020). Afterwards, the FRAM models were visualized in Adobe Illustrator 2025 (version 29). One FRAM model was made for work-as-imagined. A description of the functions included in this FRAM model is provided in Supplementary Table 2). For work-as-done, initially one FRAM model was constructed for each included clinic. After that, we combined the work-as-done FRAM models if processes overlapped. Barriers were categorized using the framework developed by Cabana et al. [21] and facilitators/novel practices were described based on data-driven analysis.

## Results

A total of four university and four community hospitals participated, with 19 interviews conducted among 14 clinicians. An overview of the participating clinics is presented in Table 1. In total, four hospitals (hospital B, C, D, and E) have established their own care pathway to conduct CV assessments, three of which are university medical centers (hospital B, C and E). For the first round of interviews, the duration of interviews ranged between 40 and 60 min. For the second interview round, when applicable, the duration of the interviews ranged between 15 and 30 min. Data saturation was achieved for both work-as-done and the identification of barriers regarding implementation of CV fall risk assessment.

**Table 1:** Overview of participating fall clinics

	University/community hospital^a^	Number of participants	Process when (pre)syncope is suspected after the initial evaluation of CV risk factors	FRAM model work-as-done variant
A	University	2	Refer to outpatient syncope clinic (cardiology)	1
B	University	1	Further CV assessments performed by geriatrician	2
C	University	2	Further CV assessments performed by geriatrician	2
D	Community	2	Fall and syncope day clinic with geriatrician as main practitioner	3
E	University	1	Further CV assessments performed by geriatrician	2
F	Community	2	Consult cardiologist	4
G	Community	2	Consult cardiologist	4
H	Community	2	Refer to outpatient syncope clinic (cardiology)	1

^a^In order of interview date

*CV* cardiovascular, *FRAM* functional resonance analysis method

### Work-as-imagined model using FRAM

**Fig. 2:**
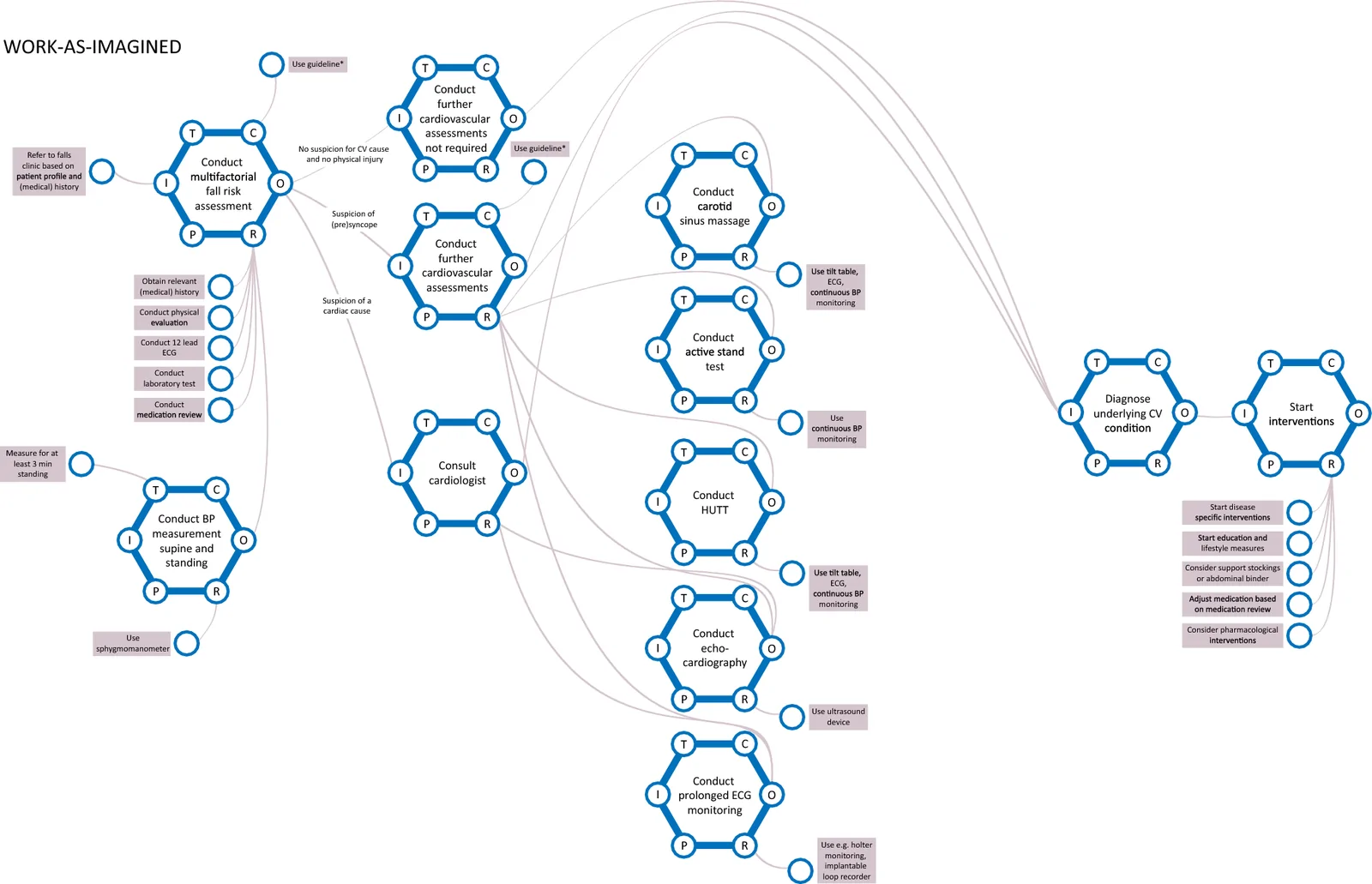
FRAM model for work-as-imagined

Figure 2 presents the FRAM model for work-as-imagined. This FRAM model consist of 33 functions, 11 of which are foreground functions. The guidelines recommendations can be divided into two main components: the initial evaluation of CV risk factors and further CV assessments. CV assessments belonging to the initial evaluation of CV risk factors are visualized as resources of the function ‘*Conduct multifactorial fall risk assessment’*. Further CV assessments are displayed across six foreground functions: consult cardiologist, conduct echocardiography, conduct prolonged ECG monitoring, conduct HUTT, conduct carotid sinus massage, and conduct active stand test. A detailed description of the foreground functions can be found in Table S1 of the supplementary data.

The 12 full functions (depicted as hexagons) represent the foreground functions. The 20 background functions are visualized as circles and have only an output (O) within the process under study. The functions are connected by lines, illustrating the flow of information or influence. For example, the output of the background function ‘refer to falls clinic based on patient profile and (medical) history’ feeds into the input of the foreground function ‘conduct multifactorial fall risk assessment’. ECG = electrocardiogram, BP = blood pressure, HUTT = head-up tilt test, CV = cardiovascular. * Dutch guidelines on fall prevention, which follows the ESC syncope guideline recommendations.

### Differences between work-as-imagined and work-as-done

For the initial evaluation of CV fall risk factors, we found complete (100%) overlap between work-as-imagined and work-as-done, indicating that all recommended CV assessments associated with the initial evaluation of CV fall risk factors were implemented in the multifactorial fall risk assessments across the participating clinics. Between fall clinics, we did find variation between protocols for several assessments included in the initial evaluation of CV fall risk factors, particularly for OH diagnostics (function: conduct blood pressure measurement supine and standing). The protocols differed among clinics in terms of standing duration (e.g., 3, 5, or 10 min) and the timing of blood pressure measurements (e.g., measuring blood pressure every minute or only at the 1st and 3rd minute standing).

Differences between work-as-imagined and work-as-done were mainly identified for the further CV assessments, which follows after the multifactorial fall risk assessment. In Table 1, the process followed when a possible (pre) syncope is suspected after the initial assessment, is summarized. In total, four different variants for the work-as-done FRAM model were identified. Variant 3 differs from the other variants in terms of patient inflow. This variant consist of a multidisciplinary care pathway, especially designed for patients with already suspected (pre)syncope. Therefore mainly patients are referred to this clinic in case of unexplained falls and/or transient loss of consciousness (TLOC).

The functions ‘*Refer to outpatient syncope clinic’, ‘Conduct twenty-four-hour ambulatory blood pressure monitoring (24-h ABPM)’*,*’Conduct postprandial blood pressure measurements’, Read out data of 24-h Holter’* and ‘*Perform follow-up to re-evaluate interventions*’ were identified as new functions and added to the corresponding work-as-done FRAM variants. After the multifactorial fall risk assessment or after further CV assessments, the functions ‘*Diagnose underlying CV condition*’ and ‘*Start interventions*’ follow in all four variants and are all in line with work-as-imagined.

### Work-as-done model for variant 1 using FRAM

**Fig. 3:**
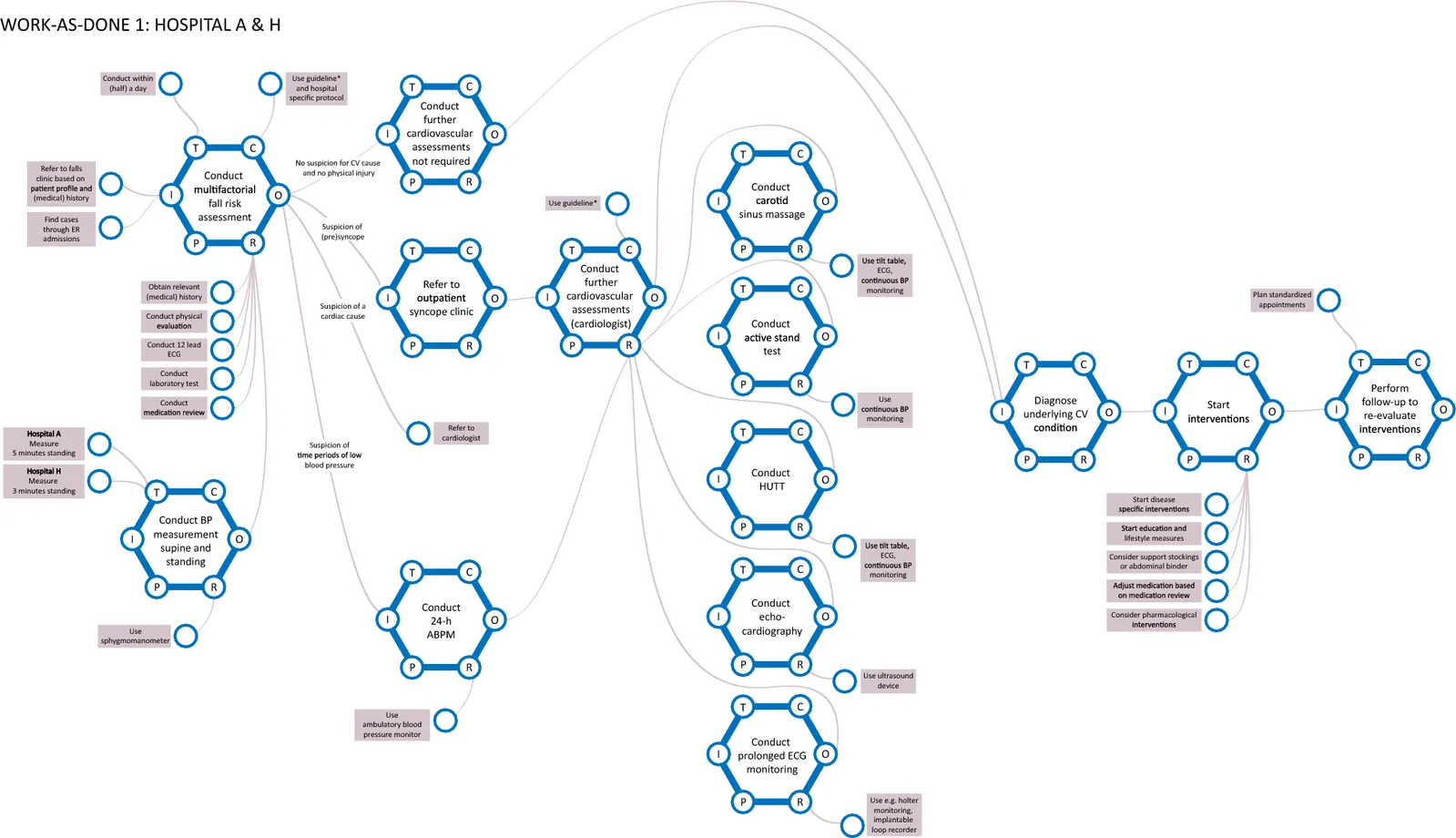
FRAM model for work-as-done variant 1. *ECG* electrocardiogram, *BP* blood pressure, *HUTT* head-up tilt test, *CV* cardiovascular, *ABPM* ambulatory blood pressure monitoring. *Dutch guidelines on fall prevention, which follows the ESC syncope guideline recommendations

The work-as-done FRAM model for variant 1 is shown in Fig. 3. The work-as-done model demonstrates complete (100%) overlap with work-as-imagined concerning the recommended further CV assessment. In variant 1, referral to the outpatient syncope clinic, located at the cardiology department, is required for the further CV assessments. Additionally, the function ‘*Conduct 24-h ABPM’* was identified as a foreground function. This function can either be performed within the syncope clinic or can be directly initiated by the geriatrician following the multifactorial fall risk assessment.

### Work-as-done model for variant 2 using FRAM

The work-as-done FRAM model for variant 2 is shown in Fig. 1 of the supplementary data section. This work-as-done model demonstrates an 85–100% overlap with work-as-imagined concerning the recommended further CV assessments. The clinics included in variant 2 have their own resources, protocols and staff, which allow the CV assessments to be carried out within the geriatrics department. All three clinics in variant 2 have included ‘*Conduct HUTT’* and ‘*Conduct carotid sinus massage*’ as standard procedures when a (pre)syncope is suspected. Two (2/3) clinics also included the active stand procedure as a standard assessment. One (1/3) of these clinics has further extended the active stand procedure by incorporating a standing duration of 10 min and head turning movements as additional components. ‘*Conduct 24-h ABPM’* was identified as an additional assessment for all three clinics, and two (2/3) clinics have the option for postprandial blood pressure measurements (function ‘*Conduct postprandial blood pressure measurements’)*. For the functions ‘*Conduct echocardiography*’ and ‘*Conduct prolonged ECG monitoring*’, referral to a cardiologist is required in these clinics.

### Work-as-done model for variant 3 using FRAM

The work-as-done FRAM model for variant 3 is shown in Fig. 2 of the supplementary data section. In variant 3, older adults with unexplained falls and/or TLOC are referred to the fall and syncope day clinic. In this multidisciplinary care pathway, patients are evaluated in 2 days, resulting in the functions ‘*Conduct multifactorial assessment (part 1)*’ and ‘*Conduct multifactorial assessment (part 2)*’. Most of the assessments belonging to the initial evaluation of CV fall risk factors are performed on the 1st day (part 1). For the recommended further CV assessments there is 57% overlap with work-as-imagined. ‘*Prolonged ECG monitoring (24-h Holter)*’ is a standard procedure performed on day 1, with the results evaluated in part 2 (‘*Read out data of 24-h Holter’).* A supine and standing blood pressure measurement is performed both in part 1 and in part 2, but with different standing duration time (5 vs 10 min). The cardiologist is routinely consulted during part 2 of the assessment. ‘*Conduct postprandial blood pressure measurement*’ was identified as an additional foreground function, belonging to part 2 of the assessment. The active stand procedure and HUTT are missing in variant 3.

### Work-as-done for variant 4 using FRAM

The work-as-done FRAM model for variant 4 is shown in Fig. 3 of the supplementary data section. The work-as-done model demonstrates 50% overlap with work-as-imagined concerning the recommended further CV assessments. In variant 4, patients are referred to a cardiologist when further CV assessments are indicated after the multifactorial fall risk assessment. The clinics in variant 4 do not have a formal syncope clinic, and the use of further CV assessments depends on the cardiologist’s consultation and the resources available at the hospital. *‘Conduct echocardiography’* and ‘*Conduct prolonged ECG monitoring*’ were added as resources belonging to the further CV assessments, and are performed by a cardiologist. For the other assessments (e.g., *Conduct HUTT*), however, information was insufficient to include these functions in the model. The clinics in variant 4 do facilitate postprandial blood pressure measurements and 24-h ABPM, leading to the inclusion of the corresponding functions in the model.

### Barriers to following work-as-imagined

Table 2 lists the barriers for following work-as-imagined mentioned by two or more participants. The full version of the table can be found in Supplementary Table 3. The identified barriers could be categorized into the following: environmental factors, guidelines-related factors, knowledge, lack of self-efficacy, and patient-related factors.

**Table 2:** Barriers to follow work-as-imagined

Barrier	Barrier description (n/n)	Quotation
Environmental factors		
Staff	Lack of staff (7/14)	*“I believe that the main obstacle may not be acquiring the equipment, but the availability of staff to carry it out. At present, this is what I see as the most significant barrier.”*
	Lack of staff with expertise (10/14)	*“In my absence, there is no one else qualified to perform the assessments, which can be become a problem during prolonged absence or unavailability. “* *“We have a continuous blood pressure monitoring option, but no trained staff”*
Lack of resources	Missing equipment (2/14)	***“****Knowledge, education and support is needed. But also the equipment, because for example a tilt table is not available in every hospital.”*
Lack of time	Time constraints (4/14)	*“Given the current time constraints, it is not feasible to perform additional assessments. It would require to schedule extra follow-up visits for patients, which does not align with the existing structure of our fall clinic.”*
	Time-consuming assessments (7/14)	*“The procedure requires two people and it takes at least 60 min. Its time-consuming nature is a significant limitation.”* *“It also requires your personal time. To perform it properly, you need to supervise closely and be present throughout the procedure, which means managing your own time constraints as well.”*
Lack of financial resources	Expensive acquisition (7/14)	*“I would like our department to have a continuous blood pressure monitor. We previously attempted to purchase one, but it was too expensive.”*
Organizational constraints	Location of resources (2/14)	*“Logistics, in particular, significantly influences the threshold for scheduling certain assessments. Since our department relocated, I have observed a notable decrease in the number of assessments performed. This decline is not due to a change in patient population, but is likely related to availability constraints. When there is clinical indication, the assessment should be conducted. Although it still occurs, it happens less frequently than before.”*
Knowledge		
Lack of knowledge	Equipment (9/14)	“*The equipment is vulnerable and therefore should not be operated by an intern without appropriate supervision."* “*In our clinic, internships last only three months, which makes it difficult for interns to perform the procedure independently. While they may occasionally observe and attempt to gain some experience, it is important to recognize that many will likely start working in settings where they will rarely, if ever, perform this procedure again.”*
	Expertise on the topic (5/14)	*“ We are working with rotating schedules, and not all staff have the same level of interest in falls. While supervision can be provided by someone less engaged, conducting these assessments requires specific experience and expertise. Scheduling challenges often hinder effective implementation.”* *“ I am not the only supervisor, and the level of knowledge on this specific topic varies among supervisors,. Therefore, I believe that not all supervisors possess sufficient expertise.”*

*N* number of participants

Barriers related to the category staff were most frequently mentioned. Seven participants reported barriers related to a general lack of staff (e.g., to support the assessments), and ten participants mentioned barriers regarding lack of staff with specific expertise. Additionally, we found that in clinics where staff with expertise was present, this expertise was often limited to a single individual, which made the execution of the assessments vulnerable in case of dropout of this individual. Nine participants reported barriers regarding lack of knowledge on procedures and equipment, and five participants mentioned a lack of knowledge regarding expertise on the topic. For most of the recommended CV assessments, experience and training for the required equipment is needed (e.g., how to use a continuous blood pressure monitor). Time constraints were identified as a barrier to conducting additional assessments by four participants, and seven participants reported barriers related to their own time availability. In general, the recommended CV assessments (e.g., HUTT) are time-consuming and do not align with their current structure of the fall clinic. Seven participants also mentioned a lack of financial resources as a barrier.

Two participants mentioned barriers related to organizational aspects. For example, in one clinic from work-as-done variant 2, the geriatrics department possessed the required resources and expertise. However, following an in-hospital reorganization, these resources were transferred to another department, leaving geriatricians dependent on the schedule and support staff of this new department. This made it more difficult to schedule the CV assessments, potentially raising the threshold for their utilization.

Two participants mentioned barriers related to patient characteristics, noting that compliance among the patient group (older adults) is generally low, and considerable motivational effort is required to encourage participation for additional assessments.

### Potential facilitators

The implementation of a standardized approach to history taking focused on CV-related falls and the integration of a Clinical Decision Support System (CDSS) were identified as potential facilitators to enhance knowledge and awareness for CV fall risk factors. Additionally, instruments and tools were mentioned as potentially valuable within the CV fall risk assessment. Near-infrared spectroscopy (NIRS) was suggested as a promising alternative to traditional blood pressure measurements regarding OH-diagnostics. Furthermore, eHealth strategies, such as a smartwatch application for the detection of clinically relevant atrial fibrillation, was mentioned as a promising tool.

Six participants (6/14) indicated that a (standardized) multidisciplinary consultation with other syncope specialists, such as cardiologists and neurologists, would be a valuable addition to a CV fall risk assessment care pathway. Furthermore, 12 (12/14) participants agreed that having a care pathway for CV-related falls could add value to fall risk evaluation, particularly in creating a more consistent policy nationwide. As one expert noted, *“ A more uniform policy is needed throughout the country, because in geriatrics there are few clinics that perform the risk assessment and management systematically. Some clinics perform comprehensive evaluations, whereas others conduct only minimal assessments.”*

Additionally, it was mentioned that a care pathway could facilitate making a business case for implementing certain CV assessments:* “In cases where a particular need arises, the guideline can serve as evidence that recommended practices are not being followed. This forms the basis for developing a business case that can be presented to the hospital board, highlighting that although the guidelines require this action, it cannot be implemented under current conditions.”*

## Discussion

In this qualitative study, we used the FRAM methodology to visualize the CV fall risk assessment across Dutch fall clinics, aiming to understand discrepancies between guidelines recommendations (work-as-imagined) and clinical practice (work-as-done). We found that work-as-done for the initial evaluation of CV risk factors was in line with work-as-imagined and implemented across all participating fall clinics. For the guidelines-recommended further CV assessments, however, we identified substantial differences between work-as-imagined and work-as-done. Specifically, we distinguished four distinct variants for the execution of the further CV assessments. The variations were driven largely by the following barriers in guidelines implementation: unavailability of staff, gaps in knowledge, lack of expertise, and lack of resources.

To the best of our knowledge, we are the first assessing in detail the implementation of CV fall risk assessment within the context of a multifactorial fall risk evaluation. A recent European survey conducted across 34 countries assessed, among other factors, current practices for fall prevention. This survey found that CV assessments, as part of a multifactorial fall risk assessment, ranked in the middle in comparison with other components in both the frequency of performance and the perceived importance of conducting them, indicating significant practice variation in Europe, in line with our findings [22]. The specific CV components included in this assessment were however not further explored in this survey.

We found that the recommended assessments from the initial CV evaluation were fully implemented and integrated into the multifactorial fall risk assessment protocols across all participating fall clinics. However, variability between protocols was observed for certain assessments, particularly in the diagnostic approach to OH. This is in line with discrepancies present between different current guidelines: the Dutch fall prevention guidelines and the ESC syncope guidelines recommend measuring blood pressure in both (resting) supine and standing position for a duration of 3 min to assess OH [5, 16]. In contrast, the World Guidelines for fall prevention and management, published in 2022, recommend blood pressure measurements at 1-min intervals, up to a total of 5 min [1]. Because OH is the most prevalent and well-established CV risk factor for falls [4, 23], a standardized and clear diagnostic protocol for OH is essential for fall prevention. Implementing the most recent recommendations could help reduce protocol variability and improve clinical practice regarding OH diagnostics in fall clinics.

Additionally, we found that some participating clinics had incorporated a protocol for postprandial hypotension (PPH) diagnostics, despite the absence of specific recommendations for its assessments within fall risk evaluation in current (inter)national guidelines [5, 16]. PPH has been associated with both falls and syncope and is notably prevalent among older adults, with recent studies reporting a prevalence of up to 40% [24]. Given its potential clinical relevance, a more clearly defined approach to assess PPH within the CV fall risk evaluation is warranted. A comprehensive history taking is a crucial component of PPH assessment, but in particular important in the evaluation of CV-related falls [5, 25]. It has been shown to be a highly effective and essential diagnostic tool, which is often the only assessment necessary (besides physical examination and ECG) to establish a diagnosis in patients with T-LOC [26]. However, its effectiveness depends on the clinician’s training and experience [27].

One study identified insufficient time allocated for thorough history taking, combined with a lack of knowledge or experience, as an important barrier [28]. The ESC guidelines on syncope also emphasize the importance of history-taking in its practical instructions [5]. Also, lack of knowledge was found to be an important topic in our study. Acquiring knowledge and training, for example to perform certain assessments, does typically fall outside the scope of standard postgraduate training and is, therefore, generally pursued by healthcare physicians with a specific interest in the topic. Furthermore, we did not explicitly explore if there was fear of harm for certain assessments. An initiative that aims to address these knowledge gaps is Syncopedia [29]. Developed by the Syncopedia Foundation, a nonprofit organization committed to improve medical knowledge, this initiative offers a freely accessible educational website for clinicians who want to learn about syncope. To further support clinicians, several participants in our study suggested implementing standardized protocols for history taking. A CDSS may assist in this as well. A CDSS can provide diagnostic support, has been shown to improve diagnostic accuracy, and can alert clinicians to specific protocols or prompt further testing when particular signs or indicators are present [30]. However, it is important that these CDSSs account for the challenges commonly encountered in older adults with unexplained falls, such as retrograde amnesia regarding the syncopal event and the frequent absence of eyewitness accounts [31].

Furthermore, it was noted that the current guidelines lack clear actionable recommendations, which can make implementation in clinical practice challenging. This should be addressed in future guideline development.

We identified considerable variability in the implementation of the recommended further CV assessments. The availability of staff, expertise, and resources were found to be the most significant factors influencing the execution of guidelines recommendations. This is in line with a recent systematic review by Vandervelde et al., which identified resource availability, as well as a lack of knowledge and skills, as the most frequently reported barriers to the implementation of multifactorial fall risk assessments in general [32]. Our findings further highlight the significance of these factors in managing CV-related falls. Of the four clinics that were able to conduct the further CV assessments themselves, three were university centers, suggesting that expertise and resources are primarily concentrated in academic settings. Clinics with healthcare professionals having specific expertise or access to essential resources/equipment were more likely to provide comprehensive care for patients with suspected CV fall risk factors. Whether this is clinically relevant (e.g., undertreatment of CV risk factors in clinics without these capabilities) has not been investigated in our study. However, prior research has shown that CV assessments are often performed inconsistently in patients with unexplained falls [33] and that the presence of non-CV fall risk factors can lead in clinical practice to overlooking CV causes [34]. Together with the variation we observed in how recommended CV fall risk assessments are implemented, this highlights the need for more consistent and integrated CV evaluations within fall risk assessments. A structured care pathway may help address these gaps. Previous studies have demonstrated that incorporating a structured CV evaluation within the context of fall clinics can be valuable for identifying CV-related fall risk factors [35, 36].

Most participants also recognized the additional value of implementing a structured care pathway. Barriers and potential facilitators identified in this study provide insights that can inform the development of such a care pathway and facilitate its implementation. Strategies to improve the implementation of fall prevention in general have been comprehensively outlined in a recent position paper by the European Geriatric Medicine Society Special Interest Group on Falls and Fractures [37]. The authors highlight the need for a standardized care pathway and implementation guide. In line with our findings, they emphasize that adaptation to local context is important, as factors influencing CV fall risk assessment, such as limited resources and organizational differences, vary significantly between clinics. To support a more effective approach to fall prevention and management, suggested is to engage policymakers and older adults, exploring supportive technologies, conducting ongoing research, and strengthening both undergraduate and postgraduate education. [37].

Although this study was conducted among Dutch fall clinics, several of the barriers of fall prevention that we identified (such as limited resources, insufficient training or knowledge of healthcare professionals, and time constraints) have also been reported across different European healthcare systems [22]. At the same time, organizational structures, referral pathways, and reimbursement mechanisms differ substantially between countries and are likely to influence how CV fall risk assessment and management strategies can be implemented in practice [32]. Consequently, while certain barriers may have broader international relevance, effective implementation will require adaptation to the specific healthcare context [32].

### Strengths and limitations

A strength of this study is the use of an independent parallel coding, which helps mitigate potential biases from individual coders. Additionally, the inclusion of fall clinics from both university and community hospitals provided a comprehensive view of daily practice, and data saturation was achieved for both work-as-done and the reported barriers. This study also has limitations. First, since we only assessed this in the Dutch setting, our findings may not be generalizable to other countries. Furthermore, this study focused solely on fall clinics, potentially overlooking important insights regarding the execution of further CV assessments in other settings where older adults may present with falls. Additionally, the interviews were conducted by a single researcher and a research assistant, both with medical backgrounds, raising the possibility of observer bias. In addition, participants were likely physicians with a particular interest in falls or cardiovascular-related falls, which may have introduced selection bias. As a result, the findings may overestimate how well current care aligns with the guidelines. Physicians without specific internist may experience different barriers to implementing guideline-recommended care, which could limit the generalizability of the results.

## Conclusion

This study highlights the discrepancies between the CV fall risk assessment protocols as recommended in (inter)national fall prevention guidelines and the actual practices implemented in Dutch fall clinics. Specifically, these discrepancies are most pronounced in the execution of further CV assessments. Our findings emphasize the importance of improving the implementation of CV fall risk evaluation in Dutch fall clinics. Addressing the identified barriers and potential facilitators is essential for development and implementation of a structured care pathway for CV assessment and management in older adults at risk of falls. Successful implementation would facilitate a more uniform approach to cardiovascular fall risk assessment and management across fall clinics.

## Supplementary Information

Below is the link to the electronic supplementary material.

Supplementary file1 (DOCX 1185 KB)

## Data Availability

Data can be made available upon reasonable request.
