# Supplementary material for: Evaluation of cardiovascular fall risk assessment and managment in Dutch fall clinics comparing current practice to guideline recommendations
Source: Eur Geriatr Med. 2026 Jun 2;17(4):2235–46. doi: 10.1007/s41999-026-01481-3 (PMC13562316; doi:10.1007/s41999-026-01481-3)
Supplement: Supplementary file 1 [file 41999_2026_1481_MOESM1_ESM.docx]

**Appendix S1.** COREQ (Consolidated criteria for Reporting Qualitative Research) checklist

| **Topic** | **Guide Questions/Descriptions** | **Pag.** |
| --- | --- | --- |
| **Domain 1: Research team and reflexivity** | | |
| ***Personal characteristics*** | | |
| **Interviewer/facilitator** | **Which author/s conducted the interview or focus group** | 8 |
| **Credentials** | **What were the researchers credentials?** | authors |
| **Occupation** | **What was their occupation at the time of the study?** | 8 |
| **Gender** | **Was the research male or female?** | authors |
| **Experience and training** | **What experience or training did the researcher have?** | 8 |
| *Relationship with participants* | | |
| **Relationship established** | **Was a relationship established prior to study commencement?** | 6 |
| **Participant knowledge of interviewer** | **What did the participants know about the researcher?** | 6 |
| **Interviewer characteristics** | **What characteristics were reported about the interviewer/facilitator?** | 8 |
| **Domain 2: study design** | | |
| ***Theoretical framework*** | | |
| **Methodological orientation and theory** | **What methodological orientation was stated to underpin the study?** | 6-8 |
| *Participant selection* | | |
| **Sampling** | **How were participants selected?** | 6 |
| **Method of approach** | **How were participants approached?** | 6 |
| **Sample size** | **How many participants were in the study?** | 9 |
| **Non-participation** | **How many people refused to participate or dropped out?**  **Reasons?** | N.A. |
| ***Setting*** | | |
| **Setting of data collection** | **Where was the data collected?** | 6 |
| **Presence of non-participants** | **Was anyone else present besides the participants and the researchers?** | 6 |
| **Description of sample** | **What are the important characteristics of the sample?** | 9 |
| ***Data collection*** | | |
| **Interview guide** | **Were questions, prompts, guides provided by the authors? Was it pilot tested?** | 8; Appendix S2 |
| **Repeat interviews** | **Were repeat interviews carried out? If yes, how many?** | 7-8 |
| **Audio/visual recording** | **Did the research use audio or visual recordings to collect the data?** | 8 |
| **Field notes** | **Were field notes made during and/or after the interview?** | N.A. |
| **Duration** | **What was the duration of the interviews?** | 9 |
| **Data saturation** | **Was data saturation discussed?** | 9 |
| **Transcripts returned** | **Were transcripts returned to participants for comment and/or correction?** | 8 |

| **Domain 3: analysis and findings**  *Data analysis* | | |
| --- | --- | --- |
| **Number of data coders** | **How many data coders coded the data?** | 8 |
| **Description of the coding tree** | **Did authors provide a description of the coding tree?** | 8; Table S1 |
| **Derivation of themes** | **Were themes identified in advance or derived from the data?** | 8 |
| **Software** | **What software, if applicable, was used to manage the data?** | 8 |
| **Participant checking** | **Did participants provide feedback on the findings?** | 8 |
| *Reporting* | | |
| **Quotations presented** | **Were participants quotations presented to illustrate the themes/findings? Was each quotation identified? E.g. participant number** | 15-16; Table S2 |
| **Data and findings consistent** | **Was there consistency between the data presented and the findings?** | 9-16 |
| **Clarity of major themes** | **Were major themes clearly presented in the findings?** | 9-16 |
| **Clarity of minor themes** | **Is there a description of diverse cases of discussion of minor themes?** | 9-16 |

**Appendix S2. Examples of semi-structured interview questions**

Perform multifactorial fall risk assessment

Input: How are patients referred to your fall clinic. What referral criteria does your fall clinic use?

Resources: Which cardiovascular diagnostics are included as standard in the multifactorial fall risk assessment?

Time: How much time is available to perform a multifactorial fall risk assessment?

Control: are these standard assessments executed according to the guidelines? Which guidelines are being used?

Consult cardiologist

Input: after the multifactorial fall risk evaluation, when do you directly consult the cardiologist? What are the triggers?

Time: how soon can a patient be seen by a cardiologist?

Conduct auxiliary CV assessments

Input: when there is an unexplained fall or possible syncope, after the multifactorial fall risk evaluation, what are the next steps? What are the triggers for the auxiliary CV assessments?

Resources: What options does your clinic have for performing the auxiliary CV assessments?

Where are the following elements performed: Echocardiography, prolonged ECG monitoring, active standing, HUTT.

Which resources are available and where?

Control: Are the guidelines clear about which auxiliary CC diagnostics are indicated and when they are indicated?

Barriers

Do you experience barriers in the implementation of auxiliary CV assessments?

Is the implementation of the auxiliary CV diagnostics influenced by staffing level?

Is the implementation of the auxiliary CV diagnostics affected by lack of resources?

Novel practices

Are there other/additional investigations not described in the guideline, that could be promising?

Other

Do you think the guidelines are clear regarding CV-related falls?

Is there anything missing in the current situation? If yes, what are you missing?

What do you think would be the biggest improvement?

Would a care pathway be of added value within a multifactorial falls evaluation?

**Table S1.** Initial code tree

| **Initial code tree based on the functions and variables of the FRAM model for work-as-imagined.** | |
| --- | --- |
| Multifactorial fall risk assessment | Control  Input  Output  Precondition  Resource  Time |
| Conduct auxiliary CV assessments | Control  Input  Output  Precondition  Resource  Time |
| Consult cardiologist | Control  Input  Output  Precondition  Resource  Time |
| Start interventions | Control  Input  Output  Precondition  Resource  Time |

**Table S2**. Description of the main functions belonging to the work-as-imagined FRAM model.

| Function | Description |
| --- | --- |
| **Conduct multifactorial fall risk assessment** | A multifactorial fall risk assessment should include the following elements to evaluate the risk of a CV cause for a fall: history of CV symptomatology and CV history (combined in function ‘obtain relevant (medical) history’), physical evaluation, blood pressure measurement supine and standing, 12-lead electrocardiogram (ECG) and laboratory tests. These test belong to the initial evaluation of CV fall risk factors. |
| **Conduct further cardiovascular assessments not required** | When there is no suspicion for a CV cause of the fall and significant physical injury is lacking, further diagnostic testing is not recommended. This function is also initiated when the initial evaluation of CV fall risk factors resulted in a (suspected) CV cause of the fall. |
| **Consult cardiologist** | Direct referral to a cardiologist directly recommended after the initial evaluation of CV fall risk factors, when there is cardiac symptomatology, when there are cardiac diseases in the medical history or when there are ECG abnormalities. |
| **Conduct further cardiovascular assessments** | Further cardiovascular assessments should be considered when (pre) syncope is suspected after the initial evaluation of CV fall risk factors. |
| **Conduct carotid sinus massage** | Carotid sinus massage is recommended to investigate CSH. A tilt table, continuous blood pressure monitoring and ECG monitoring are required as resources. |
| **Conduct active stand test** | The active stand test is recommended to investigate the presence of initial or late OH. Continuous blood pressure monitoring is recommended as resource. |
| **Conduct HUTT** | HUTT is recommended to investigate possible sensitivity to vasovagal reflex syncope (VVS) or (initial or late) OH. A tilt table, continuous blood pressure monitoring and ECG monitoring are required as resources. |
| **Conduct prolonged ECG monitoring** | When arrhythmia is suspected, prolonged ECG monitoring, such as holter monitoring or a loop recorder (external and implantable), is recommended. |
| **Conduct echocardiography** | Echocardiography should be considered if cardiac syncope is suspected or needs to be excluded. Often consultation of a cardiologist is required. |
| **Diagnose underlying CV condition** | The output from 1) the initial evaluation of CV fall risk factors, 2) the auxiliary CV assessments or 3) the cardiologist consultation, can result in diagnosing a CV cause of the fall (e.g. OH, CSH or arrhythmia). |
| **Start interventions** | After diagnosing a CV fall risk factor, both disease specific (e.g. pacemaker in case of arrhythmia) or general interventions (e.g. lifestyle recommendations), can be initiated. |
| CV: cardiovascular, ECG: electrocardiogram, CSH: carotid sinus hypersensitivity, HUTT: head-up tilt test, VVS: vasovagal reflex syncope , OH: Orthostatic Hypotension | |

**Figure S1.** FRAM model for work-as-done variant 2

* Dutch guidelines on falls prevention, which follows the ESC syncope guideline recommendations ER=emergency room, ECG=electrocardiogram, BP=blood pressure, HUTT= head-up tilt test, CV=cardiovascular, ABPM=ambulatory blood pressure monitoring

**Figure S2.** FRAM model for work-as-done variant 3

* Dutch guidelines on falls prevention, which follows the ESC syncope guideline recommendations TLOC=transient loss of consciousness, ECG=electrocardiogram, BP=blood pressure, CV=cardiovascular, ABPM=ambulatory blood pressure monitoring

**Figure S3.** FRAM model for work-as-done variant 4

* Dutch guidelines on falls prevention, which follows the ESC syncope guideline recommendations TLOC=transient loss of consciousness, ECG=electrocardiogram, BP=blood pressure, ABPM=ambulatory blood pressure monitoring , CV=cardiovascular

**Table S3.** Complete overview of identified barriers to follow work-as-imagined

| **Barrier** | **Barrier description**  **(n/n)** | **quotation** |
| --- | --- | --- |
| **Environmental factors** | | |
| Staff | Lack of staff (7/14) | *“I believe that the main obstacle may not be acquiring the equipment, but the availability of staff to carry it out. At present, this is what I see as the most significant barrier.”* |
|  | Lack of staff with expertise (10/14) | *“In my absence, there is no one else qualified to perform the assessments, which can be become a problem during prolonged absence or unavailability. “*  *“We have a continuous blood pressure monitoring option, but no trained staff”.* |
| Lack of resources | Missing resources (2/14) | ***“****Knowledge, education and support is needed. But also the equipment, because for example a tilt table is not available in every hospital.”* |
| Lack of time | Time constraints (4/14) | *“Given the current time constraints, it is not feasible to perform additional assessments. It would require to schedule extra follow-up visits for patients, which does not align with the existing structure of our fall clinic.”* |
|  | Time consuming assessments (7/14) | *“The procedure requires two people and it takes at least 60 minutes. Its time-consuming nature is a significant limitation.”*  *“It also requires your personal time. To perform it properly, you need to supervise closely and be present throughout the procedure, which means managing your own time constraints as well.”* |
| Lack of financial resources | Expensive acquisition  (7/14) | *“I would like our department to have a continuous blood pressure monitor. We previously attempted to purchase one, but it was too expensive.”* |
|  | Lack of financial renumeration (1/14) | *“It is a relatively expensive procedure. Since it falls under the same diagnosis-treatment combination payment, performing these assessments does not generate additional reimbursement. Combined with the time investment, this has made us more selective in determining when the procedure is indicated.”* |
| Organizational constraints | Location of resources (2/14) | *“Logistics, in particular, significantly influences the threshold for scheduling certain assessments. Since our department relocated, I have observed a notable decrease in the number of assessments performed. This decline is not due to a change in patient population, but is likely related to availability constraints. When there is clinical indication, the assessment should be conducted. Although it still occurs, it happens less frequently than before.”* |
| ***Guideline-Related factors*** | | |
| Guideline characteristics | Comprehensive, lack of practical guidance (1/14) | “*The guideline is very comprehensive. I think that already reflects how complex the literature is. When you look at the interpretations they provide, the evidence is often contradictory, and even the synthesis van be confusing- so it is not always clear what I should do in clinical practice.”* |
|  | Level of evidence (1/14) | *“The disadvantage is that we have limited scientific evidence, which I consider a significant shortcoming.. We do not yet know which interventions are appropriate for which patients, and much of the guideline is based on expert opinion. Consequently, not all current practice is evidence-based, highlighting the need for further research.“* |
| ***Knowledge*** | | |
| Lack of knowledge | Resources (9/14) | “*The equipment is vulnerable and therefore should not be operated by an intern without appropriate supervision."*  “*In our clinic, internships last only three months, which makes it difficult for interns to perform the procedure independently. While they may occasionally observe and attempt to gain some experience, it is important to recognize that many will likely start working in settings where they will rarely, if ever, perform this procedure again.”.* |
|  | Expertise on the topic (5/14) | *“ We are working with rotating schedules, and not all staff have the same level of interest in falls. While supervision can be provided by someone less engaged, conducting these assessments requires specific experience and expertise. Scheduling challenges often hinder effective implementation.”*  *“ I am not the only supervisor, and the level of knowledge on this specific topic varies among supervisors. Therefore, I believe that not all supervisors possess sufficient expertise.”* |
| ***Lack of self-efficacy*** | | |
|  | Supervision by an expert (1/14) | *“In our center, I feel that supervision of older adults presenting with falls should be carried out by the designated expert in our department. Since I am not that expert, I do not consider myself the appropriate person for this responsibility, although I am still required to provide supervision..”* |
| ***Patient-related factors*** | | |
|  | Patient characteristics (2/14) | *” Patient compliance could certainly improve. This is a difficult patient group, while they can quite compliant at times, they also require considerable motivational effort to encourage participation.”* |
|  | Low patient numbers (1/14) | *“We do not wish to conduct these assessments on our own. Considering the current volume of patients with a possible indication, it does not feel feasible or appropriate..”* |
|  | Topic not appealing (1/14) | *“There is limited interest in syncope/cardiovascular related falls among hospital boards and health insurances. Despite being a relatively minor expense, it is not regarded as a high-priority topic.”* |
